# Serum sphingolipid profiling as a novel biomarker for metabolic syndrome characterization

**DOI:** 10.3389/fcvm.2022.1092331

**Published:** 2022-12-12

**Authors:** Loni Berkowitz, Cristian Salazar, Carol D. Ryff, Christopher L. Coe, Attilio Rigotti

**Affiliations:** ^1^Center of Molecular Nutrition and Chronic Diseases, Department of Nutrition, Diabetes and Metabolism, School of Medicine, Pontificia Universidad Católica de Chile, Santiago, Chile; ^2^Institute on Aging, University of Wisconsin-Madison, Madison, WI, United States

**Keywords:** sphingolipids, ceramides, metabolic syndrome, inflammation, cardiovascular risk, lactosylceramide

## Abstract

**Background:**

Sphingolipids are components of cell membrane structure, but also circulate in serum and are essential mediators of many cellular functions. While ceramides have been proposed previously as a useful biomarker for cardiometabolic disease, the involvement of other sphingolipids is still controversial. The aim of this study was to investigate the cross-sectional association between blood sphingolipidomic profiles and metabolic syndrome (MetS) as well as other atherosclerotic risk factors in a large population-based study in the U.S.

**Methods:**

Clinical data and serum sphingolipidomic profiling from 2,063 subjects who participated in the biomarker project of the Midlife in the United States (MIDUS) study were used.

**Results:**

Consistent with previous reports, we found a positive association between most ceramide levels and obesity, atherogenic dyslipidemia, impaired glucose metabolism, and MetS prevalence. In contrast, most simple β-glycosphingolipids (i.e., hexosylceramides and lactosylceramides) were inversely associated with dysmetabolic biomarkers. However, this latter sphingolipid class showed a positive link with inflammatory and vascular damage-associated biomarkers in subjects with MetS. Through metabolic network analysis, we found that the relationship between ceramides and simple β-glycosphingolipids differed significantly not only according to MetS status, but also with respect to the participants' C-reactive protein levels.

**Conclusion:**

Our findings suggest that a comprehensive sphingolipid profile is more informative about MetS than ceramides alone, and it may reveal new insights into the pathophysiology and further diabetic vs. cardiovascular risk in patients with MetS.

## Introduction

Atherosclerotic cardiovascular diseases (ASCVD) are still a major leading cause of death worldwide (1). Several risk factors have been well-established, including family history, obesity, high blood pressure, dyslipidemia, type 2 diabetes, proinflammatory processes, and metabolic syndrome (MetS) (1). MetS is typically identified by several disease-related risk factors (i.e., abdominal obesity, high serum triglyceride and glucose, hypertension, and low HDL cholesterol (HDL-c) (2) and meeting these criteria increases risk for ASCVD and type 2 diabetes by two- and five-fold, respectively (3).

Patients with MetS show several lipid abnormalities beyond the well-known proatherogenic lipid profile (increased triglycerides and low HDL-c), including high levels of small, dense and modified-LDL particles (4). In fact, traditional biochemical measures alone do not explain the complexity of metabolic abnormalities associated with MetS or its related atherosclerotic cardiovascular risk (CVR) vs. diabetic risk (4). Thus, there is a lingering need to delineate other lipid biomarkers that may contribute to improve evaluation, prognosis, and treatment of this syndrome and its long-term complications.

Based on technological advances in lipidomics, researchers have identified sphingolipids as novel biomarkers of cardiometabolic risk (5, 6). However, many of the underlying mechanisms involved remain unclear and vary depending on the specific class, or even species.

**Figure 1:**
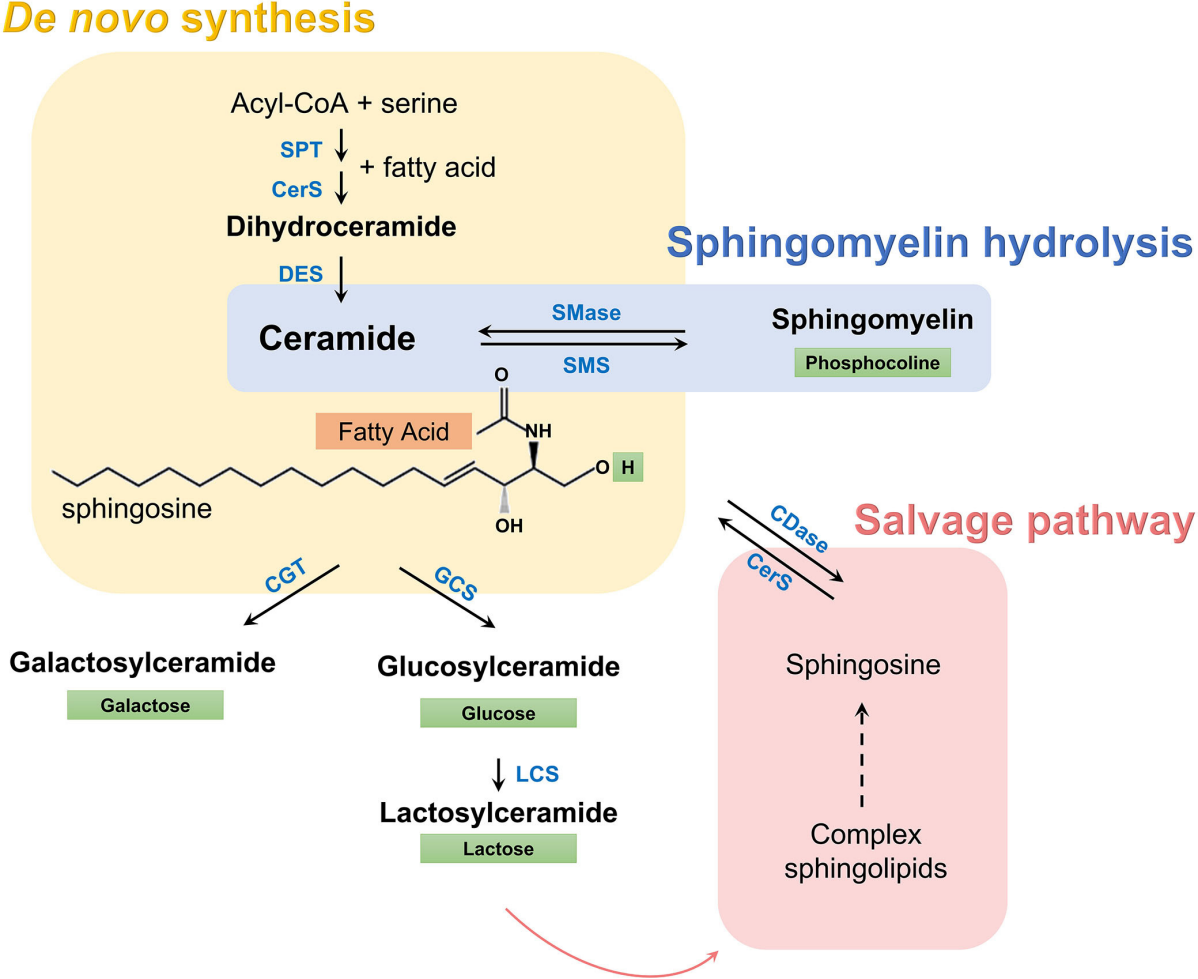
Sphingolipid metabolic pathway. A set of enzymes catalyzes the condensation of serine and acyl-CoA (usually palmitate) and *de novo* synthesis of dihydroceramides (yellow box). Dihydroceramides are then oxidized to ceramides, which are characterized by a long sphingosine carbon backbone (in black) and a fatty acid of variable length (orange box) that defines the species, according to the number of carbon atoms and double bonds. Subsequently, a hydrogen in ceramides can be replaced by a polar group, given rise to more complex sphingolipids. Depending on this polar group (green box) different classes exist, such as sphingomyelins, simple hexosylceramides (i.e., glucosylceramides and galactosylceramides), lactosylceramides, and other complex glycosphingolipids. These complex sphingolipids can be catabolized and recycled to regenerate ceramide through the salvage pathway (red box), while sphingomyelin can be hydrolyzed directly to ceramide (blue box). SPT, serine palmitoyltransferase; CerS, ceramide synthase; DES, dihydroceramide desaturase; SMase, sphingomyelinase; SMS, sphingomyelin synthase; CGT, ceramide galactosyltransferase; GCS, glucosylceramide synthase; LCS, lactosylceramide synthase, CDase, ceramidase.

Sphingolipid metabolism can be characterized as a group of metabolically connected pathways all of which converge toward ceramides (Figure 1). A substantial literature now shows that some species of ceramides may have a causative and pathogenic role in diabetes and other cardiometabolic disorders (7–12). On the other hand, the role of simple glycosylated ceramides, such as hexosylceramides and lactosylceramides, in cardiometabolic disease is unclear. While administration of these glycosphingolipids has shown remarkable beneficial effects on glucose intolerance, metabolic syndrome, and hepatic steatosis in mouse models (13–15), recent studies suggest that high levels of simple β-glycosphingolipids may increase the risk of cardiovascular events in humans (5, 16). Thus, the association of blood sphingolipid levels with MetS features may potentially reveal significant insights into the underlying mechanisms and evolution of cardiometabolic diseases in these subjects (17).

The current study investigated the relationship between blood sphingolipids and various parameters of MetS and other atherosclerotic risk factors in a large population-based study from U.S. We hypothesized that distinctive sphingolipidome profiles would be evident in adults with MetS and might vary depending on whether they exhibit a predominantly dysmetabolic pattern (i.e., obesity, dyslipidemia, and impaired glucose metabolism) or indicate more pro-inflammatory activity (i.e., high levels of pro-inflammatory proteins and endothelial dysfunction).

## Methods

### Study sample

Blood samples for the current analysis were collected as part of the Midlife in the United States (MIDUS) study, specifically from middle-aged and older adults evaluated during MIDUS 2 and MIDUS Refresher phases. MIDUS was initiated in 1995/96 to assess the role of behavioral, psychological, and social factors in adult health (18). MIDUS 2 was a longitudinal follow-up conducted approximately 9–10 years after the baseline survey and included a comprehensive biomarker evaluation of a subsample (19). The MIDUS Refresher was intended to broaden the age and racial representation by recruiting a new set of participants after the 2007–2009 great recession (20).

From subjects who participated in the biomarker project (*n* = 2,118; 1,255 from MIDUS 2 and 863 from MIDUS Refresher), we excluded 55 individuals (2.6%) who did not have lipidomic data or metabolic measures. Thus, the current analysis included 2,063 subjects (54.9% female, 74.8% white of European family backgrounds, age_*mean*_ = 55.7 ± 12.5 years) who participated in MIDUS 2 (*n* = 1,214) and MIDUS Refresher (*n* = 849) biomarker assessments. Additional descriptive information for the total sample is provided in Supplementary Table 1.

### Sociodemographic variables

Sociodemographic variables included age, gender, race, and years of education. Age was treated both as a parametric variable as well as categorical with participants in 3 age groups (<50, 50–65 and >65 years of age). Educational attainment was categorized into 3 levels: (1) high school or less, (2) some college, and (3) postgraduate studies. Race was self-reported and coded as White, African American, and others.

### Anthropometric measures and biochemical biomarkers

Health variables included body mass index (BMI, kg/m^2^), waist circumference (cm), blood pressure (mmHg), LDL-c (mg/dL), HDL-c (mg/dL), triglycerides (mg/dL), oxidized HDL (arb. unit), glycemia (mg/dL), insulinemia (μIU/mL), HbA1c (%), HOMA-IR, cytokine inflammatory markers (pg/mL), and C-reactive protein (μg/mL).

BMI, waist circumference, and blood pressure were measured by a clinical staff in one of the three Clinical and Translation Research Centers, where participants were evaluated during overnight visits (i.e., University of Wisconsin-Madison, UCLA, and Georgetown University). For this analysis, overweight was defined as >25–< 30 kg/m^2^ and BMI ≥30 kg/m^2^ was considered as obesity. In addition, information on relevant medication use was obtained at the time of blood collection.

All biochemical measures were assessed from a fasting blood sample as described elsewhere (21, 22). Briefly, a traditional lipid panel (HDL-c, estimated LDL-c, and triglyceride levels) and measures of glucoregulation (glycemia, insulinemia, HOMA-IR, and HbA1c) were determined by CLIA-certified clinical laboratories (Meriter Labs, Madison, WI and ARUP, Salt Lake City, UT). Oxidized HDL (oxHDL) levels were determined by measuring lipid peroxidation of HDL (23). Plasma fibrinogen and high sensitive C reactive protein (CRP) levels were measured using a BNII nephelometer (Dade Behring Inc., Deerfield, IL). Serum IL-6, soluble ICAM-I (soluble intercellular adhesion molecule-1), and soluble E-selectin levels were measured using high sensitivity ELISAs (R&D Systems, Minneapolis, MN).

### Serum sphingolipid profiling

Serum sphingolipid profiling was performed as part of an untargeted overall lipidomic approach by Metabolon, Inc. (Durham, NC), following the protocol described in Supplementary material (24). For the current analysis, only sphingolipid values were used, i.e., dihydroceramides, ceramides, sphingomyelins, hexosylceramides (glucosylceramides and galactosylceramides) and lactosylceramides.

Before conducting the correlational analyses, sphingolipid levels were log-transformed to achieve normal distributions and then further harmonized using z-scoring, which made the effect sizes comparable. Only those lipid species with <20% of missing values (due to being below the lower limit of detection) were included in the statistical analyses.

To calculate sphingolipid ratios, total levels of each class were used. The first ratio divides ceramide levels by lactosylceramide levels, while the second ratio partitions levels of precursor ceramides (DCER and CER) by glycosylated-ceramides (HCER and LCER). When dividing to generate sphingolipid ratios, calculations were done before log transformation.

### Metabolic syndrome

MetS was defined by the updated definition of the Adult Treatment Panel III (ATPIII) of the US National Cholesterol Education Program (25). It was based on at least three abnormal findings out of the following criteria: central adiposity (waist circumference >102 cm for men and >88 cm for women), elevated triglycerides (≥150 mg/dL) or presence of pharmacological treatment for hypertriglyceridemia, reduced HDL-c (<40 mg/dL for men and <50 mg/dL for women) or use of prescription medications to raise low HDL-c, elevated blood pressure (systolic ≥130 mmHg or diastolic ≥85 mmHg) or pharmacological treatment for hypertension in patients with a history of this disease, and high fasting glucose (≥100 mg/dL) or pharmacological treatment for hyperglycemia.

### Statistics

Participant characteristics were summarized as the mean ± SD for continuous variables or percent occurrence for categorical variables. Statistical analyses were conducted using R-studios Desktop, version 1.2.5001. All analyses were two-tailed and considered statistically significant when *p* ≤ 0.05.

*Volcano plot -* For exploratory analysis, a volcano plot was performed to visualize the overall pattern of sphingolipid species in participants with MetS. Fold change threshold was set to higher than 1.15 and statistical significance evaluated with the Wilcoxon test. For these analyses, lipid concentrations were used without any transformation.

#### Correlation analyses

All biomarker values were log-transformed to achieve graphically normal data distributions and normalized using z-scoring. Associations between sphingolipids and the health biomarkers were evaluated using linear regression models (adjusting for sex, age, race, education attainment, and MetS diagnosis) and graphed as regression coefficient matrices. Color labels represent the regression coefficients. Associations between sphingolipids and MetS components were assessed using binary logistic regression models adjusted for sex, age, race, and education attainment. Regression effects were presented as odd ratios (OR) with 95% confidence interval (CI) or graphed in a regression matrix plot. To assess the relationship between inflammatory CVR biomarkers and sphingolipid levels in subjects with or without MetS, linear regression models were used, adjusting for sociodemographic variables (sex, age, race, and education attainment).

#### Sphingolipid networks

All participants were categorized with respect to MetS criteria (positive or negative) and blood levels of CRP (below or above the median). Sphingolipid distance network analyses were performed including the sphingolipid species selected in the volcano plot and built based on the absolute Spearman correlation distance (1-|ρ|), setting to 0 those coefficients with *p* ≥ 0.05 after Benjamin-Hochberg (BH) correction. Therefore, a shorter distance represents a higher correlation magnitude between two lipid species. To identify clusters, the fast greedy modularity optimization algorithm (26) implemented in the igraph R package (27) was used to directly optimize the modularity score by merging clusters pairwise iteratively. The same method was used to analyze the networks according to alterations in glucose metabolism. For this latter analysis, impaired glucoregulation included individuals who had high glucose >100 mg/dL and insulin ≥25 mIU/L. To assess the statistical difference between complete networks, the algorithm proposed by Steiger (28) for comparison of two distance matrixes was used, implemented in the “psych” R package (29). The distance between two sphingolipid classes was calculated as the average of distances between all pairs of lipids with one species of each class. A permutation test with 1,000 repetitions was used to determine the significance of the differences in interclass distances between different groups.

### Study approval

All procedures followed were in accordance with ethical standards of the Health Sciences Institutional Review Board at the University of Wisconsin-Madison, as well as the Institutional Review Boards at the University of California-Los Angeles and Georgetown University. Informed consent was obtained from all individual participants included in the study.

## Results

### Sociodemographic, behavioral and sphingolipid differences between participants with and without MetS

Descriptive summary statistics for sociodemographic and health variables for MIDUS participants with or without MetS are provided in Table 1 (*n* = 1,292 and 773, respectively), Individuals meeting MetS criteria were older, less educated, and proportionally included more men and individuals of racial minorities. They were less likely to engage in regular exercise and more healthy diet. In addition, more than 95% of the adults with MetS were overweight or obese.

**Table 1:** Comparison of sociodemographic and health characteristics between American adults with and without metabolic syndrome (MetS).

**Characteristic**	**Frequency**		***p*-value**
	**Without MetS *n* = 1,292**	**With MetS** ***n* = 773**	
**Sex**			
Men	532 (41.2%)	399 (51.7%)	<0.001
Women	759 (58.8%)	373 (48.3%)	
**Age**			
<50 years	475 (36.7%)	218 (28.2%)	<0.001
50–65 years	546 (42.3%)	348 (45.1%)	
>65 years	270 (20.9%)	206 (26.7%)	
**Race**			
Whites	994 (76.9%)	549 (71.1%)	0.043
African Americans/Blacks	207 (16.0%)	158 (20.5%)	
Native Americans	79 (6.1%)	57 (7.4%)	
Asian	9 (0.7%)	5 (0.7%)	
Others	2 (0.2%)	3 (0.4%)	
**Educational level**			
High school or less	265 (20.5%)	222 (28.8%)	<0.001
Some college education	692 (53.6%)	385 (49.9%)	
Postgraduate studies	334 (25.9%)	165 (21.4%)	
**Nutritional status**			
Under weight	11 (0.9%)	0 (0%)	<0.001
Normal weight	490 (38.0%)	32 (4.2%)	
Overweight	328 (25.4%)	192 (24.9%)	
Obese	462 (35.8%)	548 (71.0%)	
**Regular exercise**			
No	254 (19.7%)	253 (32.8%)	<0.001
Yes	1,037 (80.3%)	519 (67.2%)	
**Smoking status**			
Never	753 (58.4%)	411 (53.3%)	0.077
Former	375 (29.1%)	252(32.7%)	
Current	161 (12.5%)	108 (14%)	
**Healthy diet index**			
≤ 6.5 (Unhealthy)	879 (68.4%)	562 (73.5%)	0.002
>6.5 (Healthy)	407 (31.6%)	203 (26.5%)	

**Figure 2:**
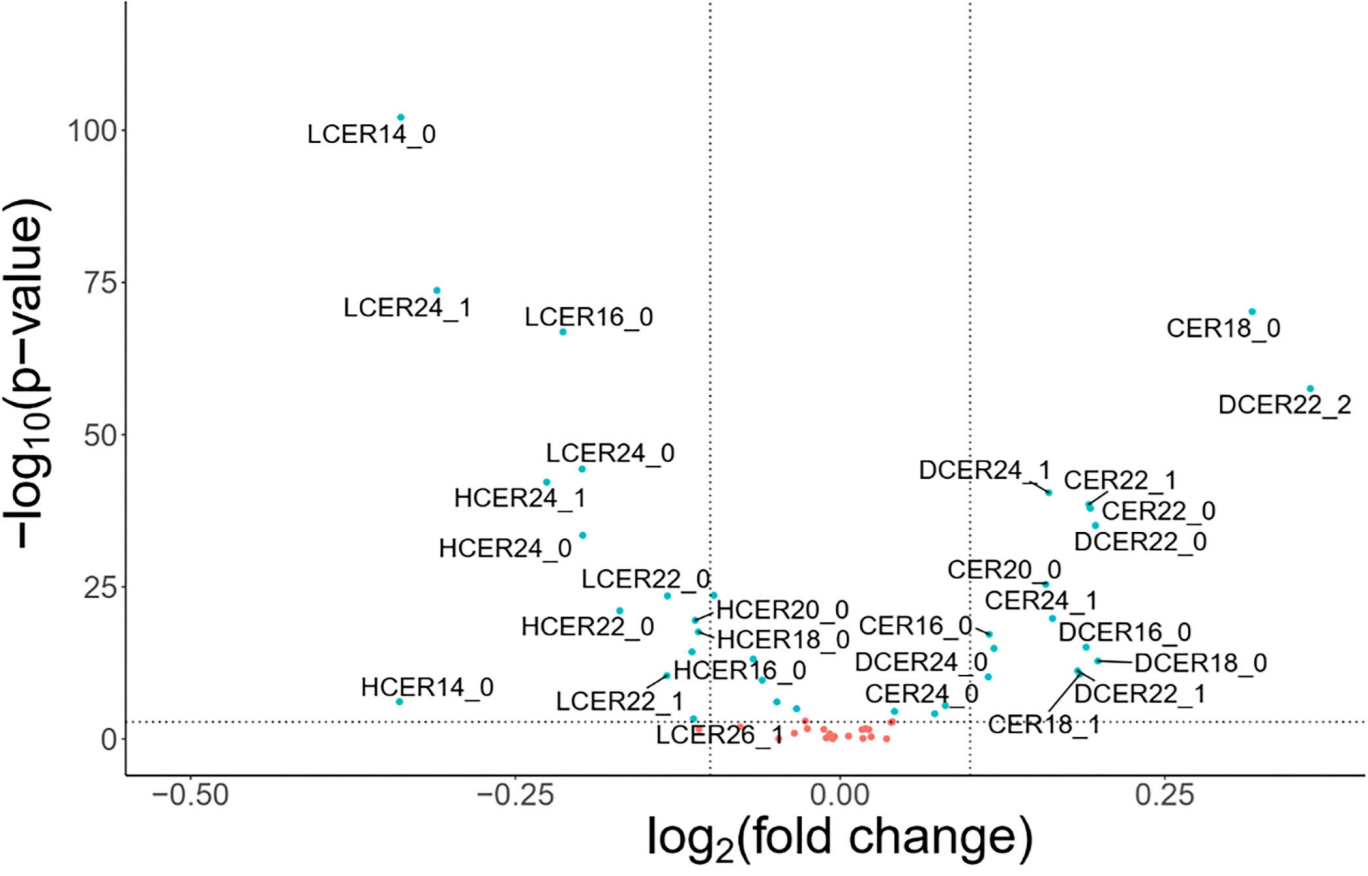
Volcano plots of sphingolipid species levels in adults with or without metabolic syndrome. Statistical significance was evaluated by Wilcoxon-test and fold change threshold in sphingolipids was set to higher than 1.15. Positive log_2_(fold change) indicates increased abundance in MetS adults and light blue circles denote *p* < 0.05. For these analyses, blood lipid levels were used without log transformation.

To explore the differences in the sphingolipid profile between adults with and without MetS, an exploratory volcano plot analysis of sphingolipid species was performed (Figure 2). Most ceramide and dihydroceramide species were higher in individuals with MetS, and increased levels of CER18 and DCER22:2 were especially prominent. On the other hand, most lactosylceramide and hexosylceramide species were lower in subjects with MetS, with LCER14, LCER24:1, and LCER16 being particularly distinctive. In contrast, there were not any significant differences in sphingomyelin species between the two groups. Based on these findings, subsequent analyses focused on ceramide, dihydroceramide, hexosylceramide, and lactosylceramide levels.

### Opposite relationships of ceramides and simple β-glycosphingolipids with MetS criteria

To analyze the association of the sphingolipid classes and MetS prevalence, binary logistic regression models were run, adjusted for sex, age, race, and education attainment.

**Figure 3:**
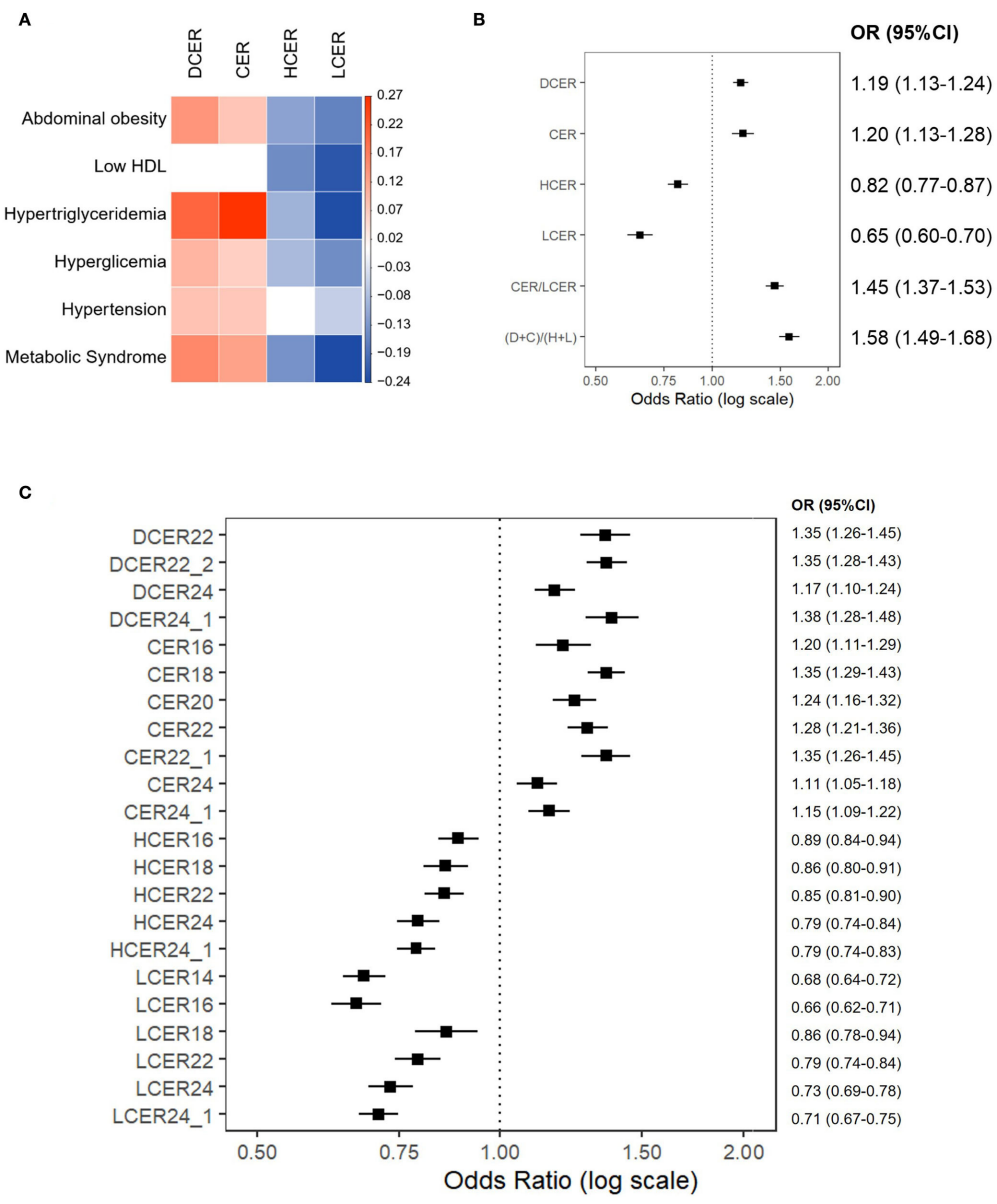
Association of blood sphingolipid profile and metabolic syndrome. Binary logistic regression models for the association between MetS and its components with blood sphingolipid levels (log-transformed), adjusted for sex, age, race, and education. **(A)** Regression coefficient matrix of the adjusted logistic regression of MetS components with blood sphingolipid levels. Color label represents the regression coefficient of those associations with p<0.05 (positive in red, and negative in blue). **(B)** Odds ratios for MetS (with 95%CI) per increase in blood levels of sphingolipid classes, as well as CER/LCER and (DCER+CER)/(HCER+LCER) ratios. **(C)** Odds ratios for MetS (with 95% CI) per increase in blood levels of sphingolipid species.

As shown in Figure 3A, dihydroceramides and ceramides were positively associated with abdominal obesity, high triglycerides, high fasting glucose, blood hypertension, and MetS. Only their association with low HDL-c was not statistically significant. In contrast, hexosylceramides and lactosylceramides had a negative association with most of these indicators. In particular, the levels of lactosylceramides revealed a strong negative association with all the MetS components.

Figure 3B shows the likelihood of meeting MetS criteria (with 95% CI) for incremental increases in blood sphingolipid levels. Based on this analysis, higher dihydroceramide levels were significantly associated with a 19% higher prevalence of MetS (OR 1.19, 95% CI 1.13–1.24). Similarly, higher ceramide levels were associated with a 20% higher prevalence of MetS (OR 1.20, 95% CI 1.13–1.28). In contrast, higher hexosylceramide and lactosylceramide levels were associated with 18% (OR 0.82, 95% CI 1.77–1.87) and 35% lower prevalence of MetS, respectively (OR 0.65, 95% CI 0.60–0.70).

Considering that association patterns of ceramides and simple β-glycosphingolipids with MetS appeared to be consistently inverse, we next examined ceramides/simple β-glycosphingolipids ratios. Based on adjusted logistic regression models, both ratios –including 1 or 2 sphingolipid classes either in the numerator or denominator– were significantly associated with a higher prevalence of MetS (Figure 3B). In particular, an integrative ratio including the four classes showed the strongest association with MetS prevalence (OR 1.58, 95% CI 1.49–1.68).

Because the level of each lipid species may differ within a sphingolipid class (30), the probability of meeting MetS criterion based on incremental increases in each sphingolipid species were evaluated. Figure 3C shows the species that revealed a significant association with MetS. In these analyses, all tested dihydroceramide species and seven out of eight ceramide species were significantly associated with a higher prevalence of MetS. Specifically, DCER22, DCER22:2, DCER24:1, CER18, and CER22:1 stood out as being strongly related to MetS. In parallel, five of seven hexosylceramides and six of seven lactosylceramides were significantly associated with a lower prevalence of MetS. The strongest negative associations were found for LCER14 and LCER16. Therefore, most ceramide and simple β-glycosphingolipid species were well represented by their total class.

### Opposite relationships of ceramides and simple β-glycosphingolipids with biomarkers of metabolic disorders

**Figure 4:**
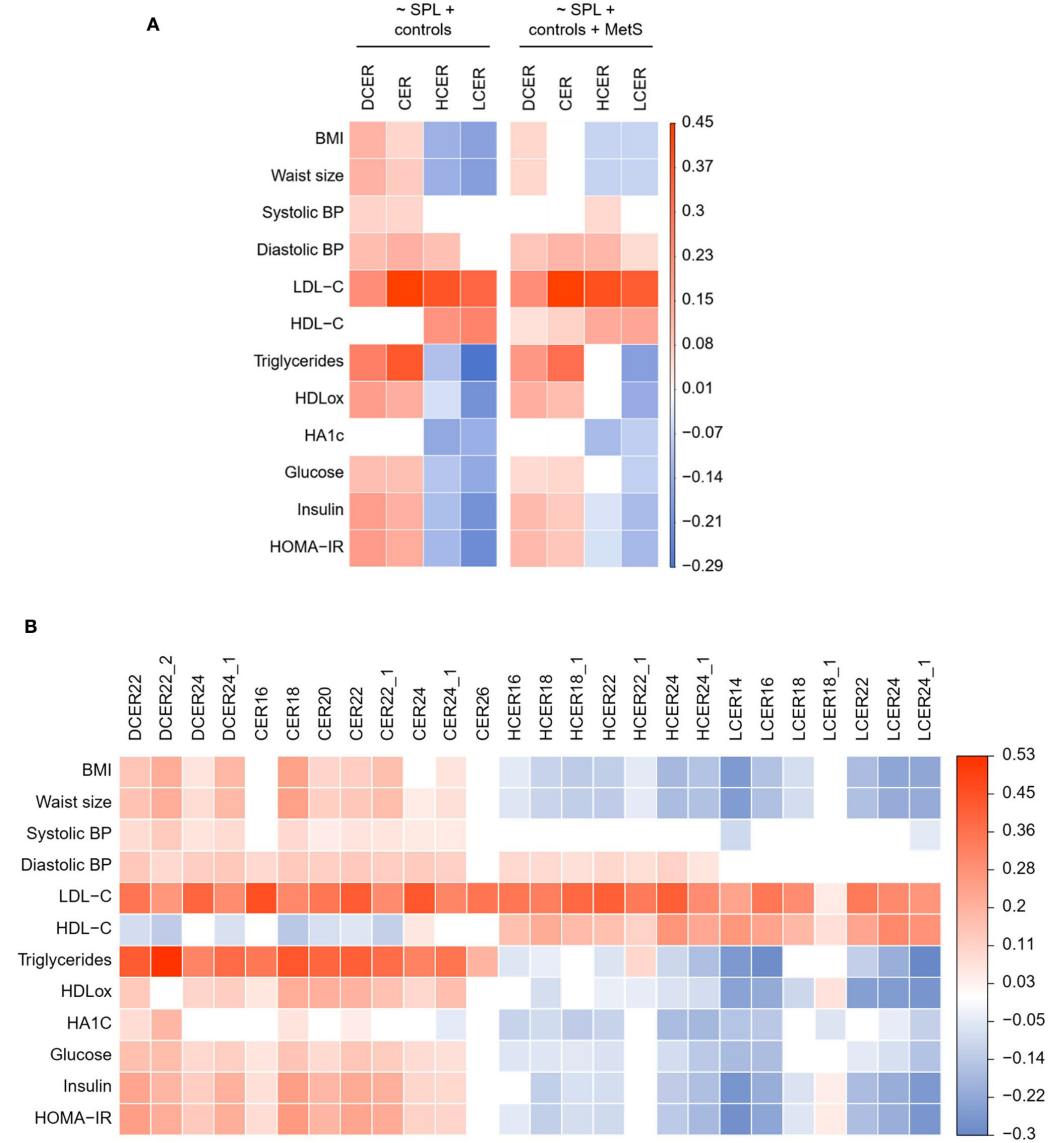
Adjusted linear regression of blood sphingolipid levels and health biomarkers. **(A)** Regression coefficient matrix of sphingolipid classes on health biormarkers adjusted for control variables (sex, age, race, and education) and MetS prevalence. **(B)** Regression coefficient matrix of sphingolipid species on health biormarkers adjusted for sex, age, race, and education. Color label represents the regression coefficient of those associations with *p* < 0.05 (positive in red, and negative in blue).

The association of blood sphingolipid levels with several metabolic biomarkers was examined with linear regression modeling adjusted for sociodemographic variables. As shown in Figure 4A, and consistent with the logistic regression, dihydroceramide and ceramide classes evinced a significant positive correlation with almost all risk biomarkers of cardiometabolic disease, including obesity, blood pressure, atherogenic lipids, and impaired glucoregulation. In contrast, hexosylceramide and lactosylceramide classes were negatively associated with most of these biomarkers, indicative of poor metabolic health. Specifically, hexosylceramide and lactosylceramide levels exhibited a significant negative association with obesity and impaired glucoregulation (Figure 4A). Moreover, lactosylceramide levels were associated with a healthier lipid profile (i.e., lower triglycerides and oxHDL, and higher HDL-c). To verify that the separate associations were not just connected to the overall MetS condition, the linear regression models were adjusted for MetS prevalence. As shown in Figure 4A (right panel), most of the individual associations remained significant in the MetS-adjusted model.

We next delineated the relationship between specific species and health biomarkers, after adjusting for sociodemographic variables (Figure 4B) and prevalence of MetS (Supplementary Figure 1). As shown in Figure 4B, the four analyzed dihydroceramide species had a significant positive relationship with obesity, blood pressure, atherogenic lipids, and impaired glucoregulation. Within the ceramides, seven of the eight analyzed species were positively associated with most of the CVR biomarkers, except for CER26 that was associated only with atherogenic lipids (LDL-c and triglycerides). With regard to the hexosylceramide species, only HCER22:1 showed a distinctive relationship with the health biomarkers (Figure 4B). While all other species were negatively associated with obesity and impaired glucose metabolism, HCER22:1 was not correlated with healthier glucoregulation. Moreover, HCER22:1 displayed a positive association with high triglyceride levels, while almost all other species were associated with a healthier lipid profile (i.e., lower triglycerides and oxHDL, and higher HDL-c). A similar pattern was evident with lactosylceramides. Six of seven species displayed a significant negative association with obesity, dyslipidemia, and impaired glucoregulation. Conversely, LCER18:1 was positively associated with some biomarkers, including oxHDL, insulin, and HOMA-IR, but not others (Figure 4B). Thus, just some lipid species of a same class behaved in a distinctive manner, and most of them were well represented by total class levels.

In addition, most associations detected for sphingolipid species remained unchanged after adding MetS as a control variable in the MetS-adjusted models (Supplementary Figure 1).

### Positive association between sphingolipid levels and inflammatory biomarkers

**Figure 5:**
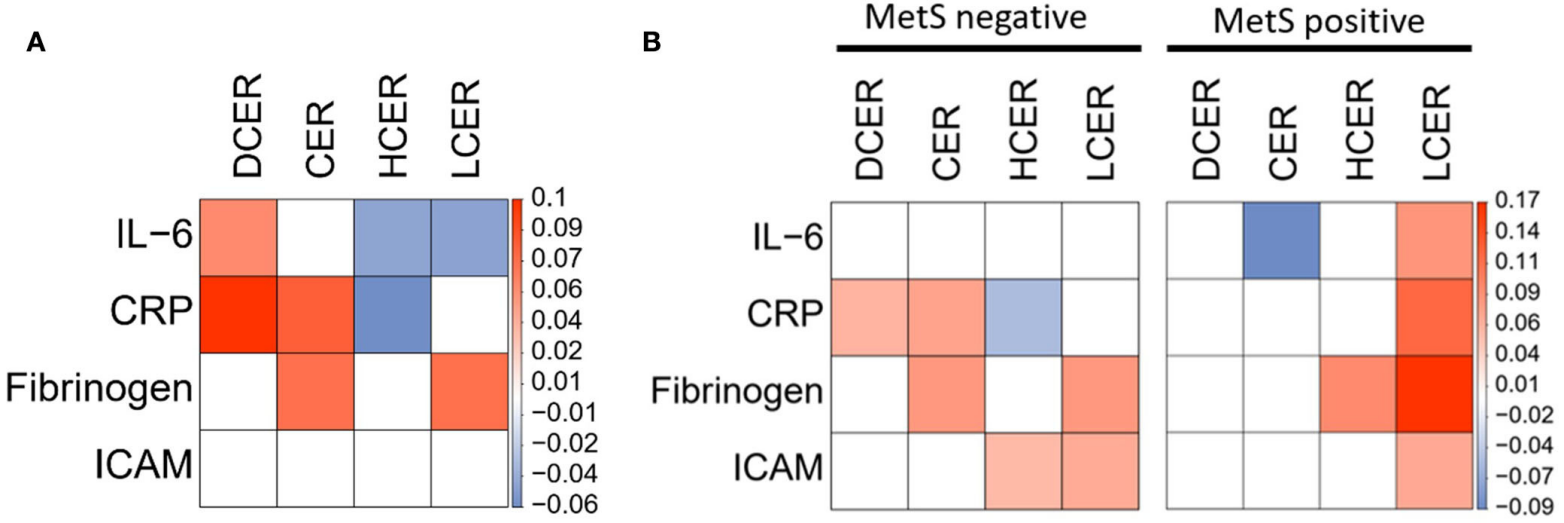
Adjusted linear regression of blood sphingolipid levels and inflammatory cardiovascular risk biomarkers. Regression coefficient matrix between sphingolipid classes and inflammatory mediators and adhesion molecules, adjusted for control variables (sex, age, race, and education) in **(A)** all subjects or **(B)** separated by absence or presence of MetS diagnosis. Color label represents the regression coefficient of those associations *p* < 0.05 (positive in red, and negative in blue).

Because inflammatory processes can also contribute to atherogenesis and ASCVD, the associations between sphingolipid levels and inflammatory mediators and adhesion proteins were assessed. Simple β-glycosphingolipids were negatively associated with inflammatory measures, including IL-6 and CRP, after adjusting for sociodemographic variables, while dihydroceramides and ceramides were positively related with most of these biomarkers (Figure 5A). Considering potential covariation with the overall MetS diagnosis, the analysis was rerun separately for participants in each subgroup. In adults without MetS, the four sphingolipid classes were still positively associated with at least one inflammatory marker (Figure 5B, left panel). Surprisingly, while no ceramide or dihydroceramide was positively related to inflammatory activity in the participants with MetS, blood levels of lactosylceramide were significantly associated with higher levels of the four inflammatory biomarkers (Figure 5B, right panel).

### Sphingolipid network differences according to MetS diagnosis and inflammatory status

**Figure 6:**
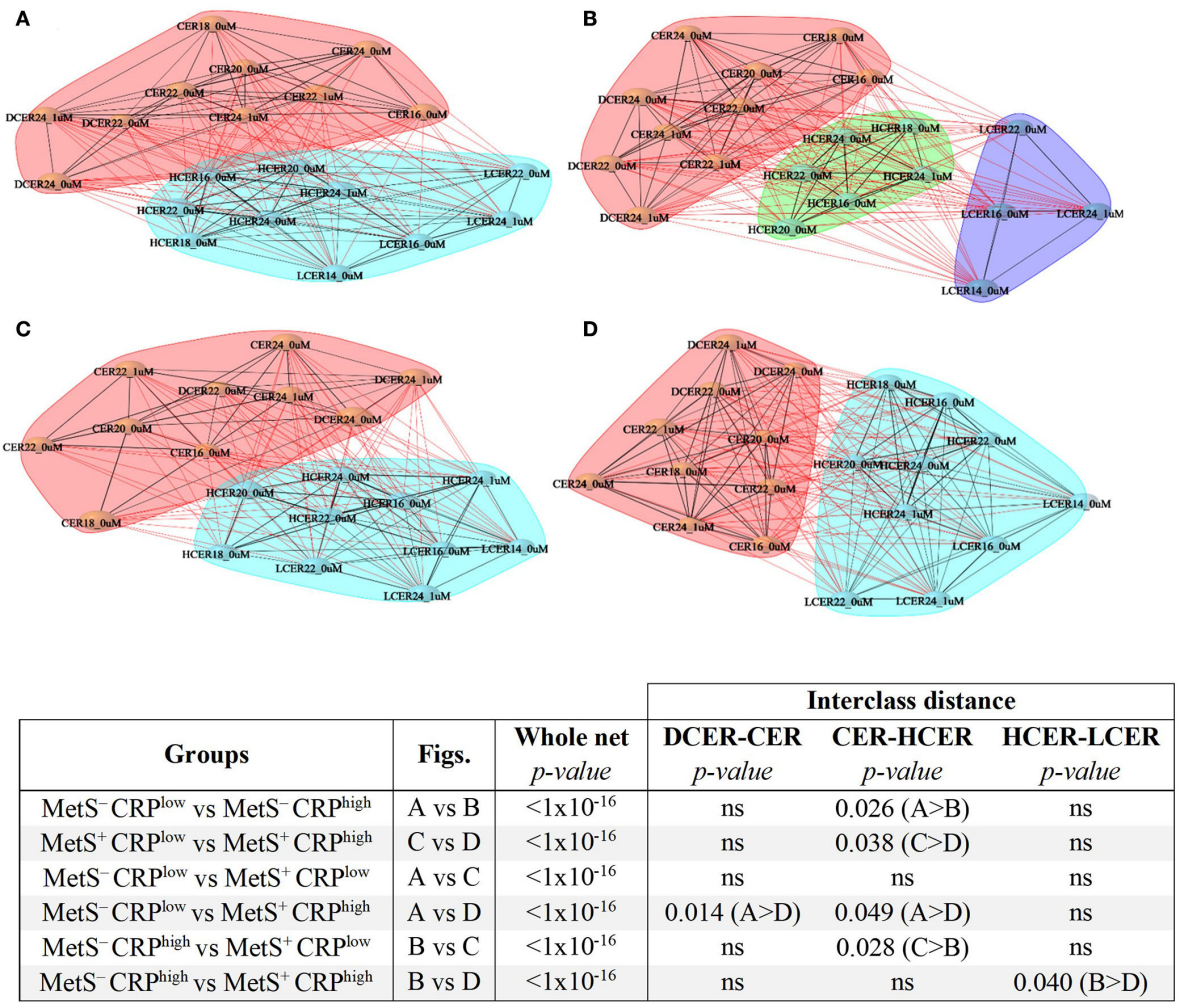
Sphingolipid distance networks based on metabolic syndrome diagnosis and CRP levels. Network analyses were performed using sphingolipids selected in the volcano plot and built based on significant correlations (BH adjusted-Spearman correlation *p* < 0.05). Nodes indicate sphingolipid species and connecting lines represent sphingolipid distances. Black lines denote intra-cluster relationships and red lines represent inter-cluster relationships. Various clusters are indicated by different colored cloud clusters. **(A)** Sphingolipid network in adults without MetS and low levels of CRP (*n* = 770). **(B)** Sphingolipid network in adults without MetS and high levels of CRP (*n* = 517). **(C)** Sphingolipid network in adults with MetS and low levels of CRP (*n* = 260). **(D)** Sphingolipid network in adults with MetS and high levels of CRP (*n* = 505). Statistical comparisons of whole networks and interclasses distances between groups are shown.

To evaluate the alterations of sphingolipid metabolic networks related to MetS and inflammatory status, distance networks were constructed and the relationship between sphingolipid species was assessed (Figure 6). For this analysis, subjects were subdivided regarding MetS criteria (i.e., positive or negative) and their CRP levels (i.e., below or above the median), and then the sphingolipid networks of the 4 subgroups were compared.

The sphingolipid networks of all subgroups displayed several broad similarities. In agreement with the previous results, ceramides and dihydroceramides formed a different cluster that was distinct from simple β-glycosphingolipids (hexosylceramides and lactosylceramides), a finding that was evident in all 4 groups (Figures 6A–D).

**Figure 7:**
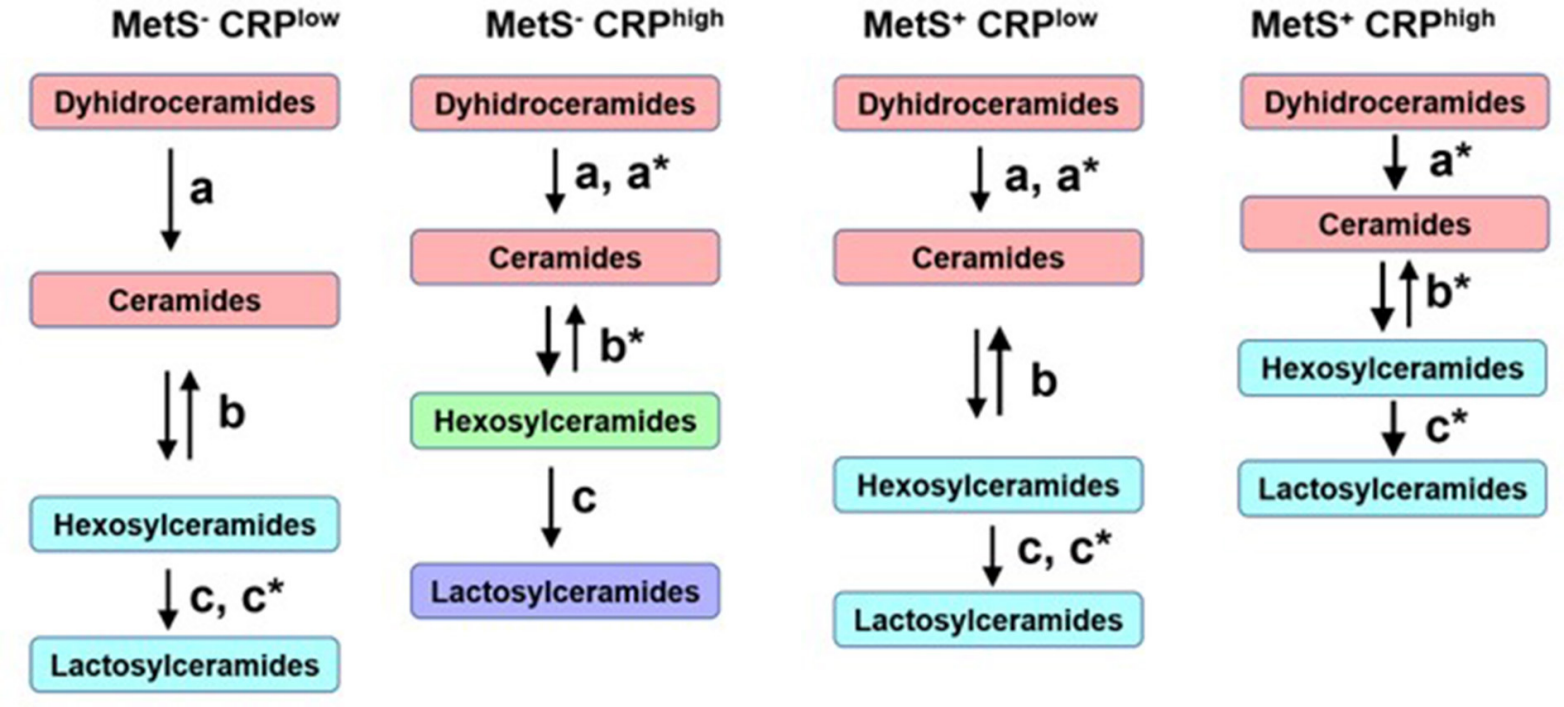
Postulated metabolic changes based on sphingolipid networks analyses and comparisons in metabolic syndrome. Different colors indicate different clusters. The length of the arrows represents interclasses distances and the letters–with or without asterisk–indicate the statistical differences obtained between the four networks.

Despite these overall similarities, there were some important differences in the networks across the 4 groups (Figure 6). Participants with low CRP who did not meet MetS criterion were considered as the reference healthier group (MetS^−^CRP^low^, Figure 6A). When the distances between sphingolipid classes were compared (Figures 6, 7), adults without MetS and high CRP levels (MetS^−^CRP^high^, Figure 6B) exhibited a stronger association between precursor ceramides and hexosylceramide levels as well as a lower correlation between hexosylceramide and lactosylceramide levels. Consequently, the distance between ceramides and hexosylceramides in MetS^−^CRP^high^ subjects was shorter, while the hexosylceramides were separated from the lactosylceramide cluster (Figure 6B). On the other hand, subjects who exhibited MetS but had low CRP levels (MetS^+^CRP^low^, Figure 6C) showed a slight strengthening of the association between dihydroceramides and ceramides, without other differences, when compared with MetS^−^CRP^low^ subjects (Figure 6A). Interestingly, when participants did have elevated CRP levels in the context of MetS (MetS^+^CRP^high^), the association between all classes was increased, which manifested by a shorter distance between them (Figure 6D). Postulated changes along the sphingolipid metabolic pathway based on interclass distances and cluster analyses are presented in Figure 7.

Finally, we determined the impact of poor glucoregulation on sphingolipid networks of participants with or without MetS (Supplementary Table 2). Network patterns were significantly different in adults showing signs of impaired glucoregulation. However, no major differences were found in inter-class distances. Only adults with impaired glucose metabolism and MetS showed a stronger association between hexosylceramides and lactosylceramides, when compared to the healthier subgroup without either condition.

## Discussion

The current analyses revealed opposite associations between ceramides and simple β-glycosphingolipid with MetS and its related metabolic, inflammatory, and endothelial dysfunction pathophysiological biomarkers. Specifically, high ceramide but low simple β-glycosphingolipid levels were linked with obesity, atherogenic dyslipidemia, and unhealthy glucoregulation. However, in contrast to their inverse relationship with metabolic markers, high levels of lactosylceramides were positively correlated with inflammatory and endothelial CVR markers in adults with MetS. These differential –even divergent– associations indicate the multifactorial aspects of lipidomic profiles and their association with disease risk factors. It also conveys how sphingolipid profiling may provide new insights into the underlying pathways that mediate metabolic vs. cardiovascular risk in MetS.

Unlike most previous lipidomic studies performed in patients with ASCVD or DM (5, 6, 16, 31), our findings allowed a detailed characterization of sphingolipid levels and the potential pathobiology that lies beneath the context of MetS. For example, if sphingolipid synthesis was globally upregulated with increasing lipid storage observed in MetS (32), all sphingolipids should have a positive correlation with obesity related disorders. The distinctive relationships between the precursor ceramides when compared to the down-stream simple β-glycosphingolipids suggests the regulation of sphingolipid metabolism and its contribution to metabolic disorders are more complex than expected. Moreover, within each sphingolipid class, a variety of species can be defined by structural and chemical features that may have different functions or targets. In this study most species behaved similarly within the same class. The species with the highest positive association to MetS were CER18 and DCER22, and those with the highest negative association were LCER14 and LCER16. These results are consistent with previous studies showing similar profiles in patients with insulin resistance or diabetes (30, 33).

Recent research indicates that visceral obesity and chronic inflammation increase dihydroceramide and ceramide levels, which in turn can worsen MetS-associated pathophysiology, including atherothrombotic status and insulin resistance, which ultimately leads to clinical manifestations of ASCVD and/or type 2 diabetes, respectively (32, 34–37). Indeed, ceramides have been identified as candidate biomarkers of ASCVD risk and mortality (9, 16, 38). However, using different methodologies and populations, three recent studies reported negative correlations between hexosylceramide levels and the biochemical and clinical measures of obesity and diabetes (6, 39, 40). Those results are consistent with our findings from a MetS perspective. Furthermore, one study found a negative association between simple β-glycosphingolipids and the diagnosis of type 2 diabetes during follow-up (6).

Therefore, the metabolic pathway of sphingolipid synthesis is not completely up-regulated in MetS or some other compensatory pathway exists. Considering that simple β-glycosphingolipids were decreased in adults with MetS and diabetes, a rise in the salvage pathway leading to the production of ceramides by catabolism of hexosylceramides and lactosylceramides may be enhanced (Figure 1).

Based on this, we propose to include simple glycosphingolipids as the denominator in a ratio between ceramides and β-glycosphingolipids, to be used as a novel metabolic and diabetic risk indicator with a better predictive value rather than the evaluation of ceramides alone (10). The use of a simple ratio of ceramides to β-glycosphingolipids allows normalization of ceramide values by other sphingolipid classes. This standardization would be particularly useful when making comparisons across racial groups, including African Americans, in whom the overall sphingolipid levels are lower than in European American subjects, who are typically used as reference populations (41, 42). Further longitudinal follow-up in MIDUS as well as validation in additional prospective cohorts should establish the actual predictive value of this sphingolipid ratio, for instance, in forecasting the incidence of type 2 diabetes.

However, this ratio of ceramide and simple β-glycosphingolipid levels would only be valid for glucometabolic alterations present in MetS that are associated with progression to diabetes. It may not necessarily apply as predictor of CVR. Indeed, two large cohort-based lipidomic studies positively associated high levels of hexosylceramides and lactosylceramides with future cardiovascular events and cardiovascular death (5, 16). However, the cross-sectional design of our analysis prevented prospective analysis on the association between simple β-glycosphingolipids and cardiovascular outcomes. Given that we have not completed a detailed follow-up on ASCVD incidence in our cohort, we evaluated the association between sphingolipid levels and pathogenically relevant inflammatory and endothelial markers as surrogates of future CVR. Consistent with previous studies (5, 16), a positive relationship between lactosylceramides with IL-6, CRP, fibrinogen, and sICAM was found in subjects with MetS, strongly suggesting that high levels of these β-glycosphingolipids may be associated with increased risk of future cardiovascular events. Indeed, correlation networks analysis showed significant differences in the sphingolipidome of individuals with or without MetS based on their CRP levels, a key biomarker related with initiation, progression, and ischemic complications of atherosclerosis (43).

Based on these findings, new hypotheses can be postulated regarding changes in sphingolipid metabolism (Figure 7) as cause and/or consequence of metabolic and inflammatory alterations in subjects with MetS. The sphingolipid cluster distance analysis suggests that high levels of CRP generate a greater imbalance in sphingolipid metabolism than the presence of MetS *per se*. High levels of CRP in the absence of MetS seems to stimulate the flux from dihydroceramides to hexosylceramides. This implies a stronger metabolic association between hexosylceramides and their precursors, rather than lactosylceramide levels downstream. In contrast, subjects with MetS and low CRP levels displayed a strengthened association between dihydroceramides and ceramides, without changes in the relationship with simple β-glycosphingolipids. The salvage pathway for ceramide formation may be activated as a compensatory mechanism, as mentioned above. Finally, the presence of MetS and high levels of CRP seems to affect the entire metabolic pathway of sphingolipids, increasing the association between all 4 classes analyzed. This means that high levels of the precursor forms would also lead to increased production of lactosylceramides, unlike what was observed in other subgroups. Therefore, the sphingolipidomic network profile may allow to differentiate subjects not only by MetS diagnosis, but also by their current inflammatory status.

Even though MetS has previously been associated with higher odds of cardiovascular events, it remains unclear whether MetS should be considered a single clinical entity or a group of different clinical conditions with diverse underlying mechanisms and carrying also distinctive cardiovascular vs. metabolic risks (44). According to our findings, more specific scores for cardiovascular vs. metabolic risk, using novel biomarkers that include both ceramides and glycosphingolipids, should allow a better stratification of MetS-associated risks.

The present study has several strengths, including the availability of lipidomic data and many clinical biomarkers in a large number of American adults. However, the cross-sectional design of this study prevents inferring causal relationships between baseline sphingolipid profiling and the progression of MetS. Moreover, cardiovascular events usually result from long-term and sustained metabolic and inflammatory conditions (45). Thus, further evidence from longitudinal studies and independent cohorts is needed to evaluate if different sphingolipid profiles -beyond ceramides- may improve prediction of progression of MetS into diabetes vs. ASCVD. Additional follow-up studies can also reveal variations in blood sphingolipid patterns along time and whether these sequential changes correlate with MetS progression to chronic disease.

In conclusion, our results showed opposite relationships of ceramides and simple β-glycosphingolipids, specially lactosylceramides, with MetS and its glucometabolic related biomarkers. Interestingly, although simple β-glycosphingolipids were associated inversely with metabolic measures, they had a positive association with inflammatory and endothelial CVR markers in subjects with MetS. Thus, our study suggests that a broader analysis of the blood sphingolipid profile, rather than ceramide levels only, may be applied as a more informative MetS-associated biomarker. In addition, it may provide new and relevant insights on the progression of this increasingly and highly prevalent risk condition that enhances the burden of chronic diseases worldwide.

## Data availability statement

Publicly available datasets were analyzed in this study. This data can be found here: https://midus.colectica.org/. Lipidomic data was deposited to OSF database under the https://doi.org/10.17605/OSF.IO/VFR7B.

## Ethics statement

The studies involving human participants were reviewed and approved by the Health Sciences Institutional Review Board at the University of Wisconsin-Madison, as well as the Institutional Review Boards at the University of California-Los Angeles and Georgetown University. The patients/participants provided their written informed consent to participate in this study.

## Author contributions

LB conducted analyses, interpreted results, and drafted the manuscript. CS assisted with data management, analysis, and manuscript revision. CC and CR participated in conception and design of the study, obtained funding, took care of the project administration, and critically reviewed the manuscript. AR was involved in conception, design of the present study, and substantial revision of the work. All authors contributed to the manuscript and approved the submitted version.
