# Supplementary material for: Serum sphingolipid profiling as a novel biomarker for metabolic syndrome characterization
Source: Front Cardiovasc Med. 2022 Dec 12;9:1092331. doi: 10.3389/fcvm.2022.1092331 (PMC9791223; doi:10.3389/fcvm.2022.1092331)
Supplement: Supplementary file 1 [file Data_Sheet_1.PDF]

Supplementary Materials

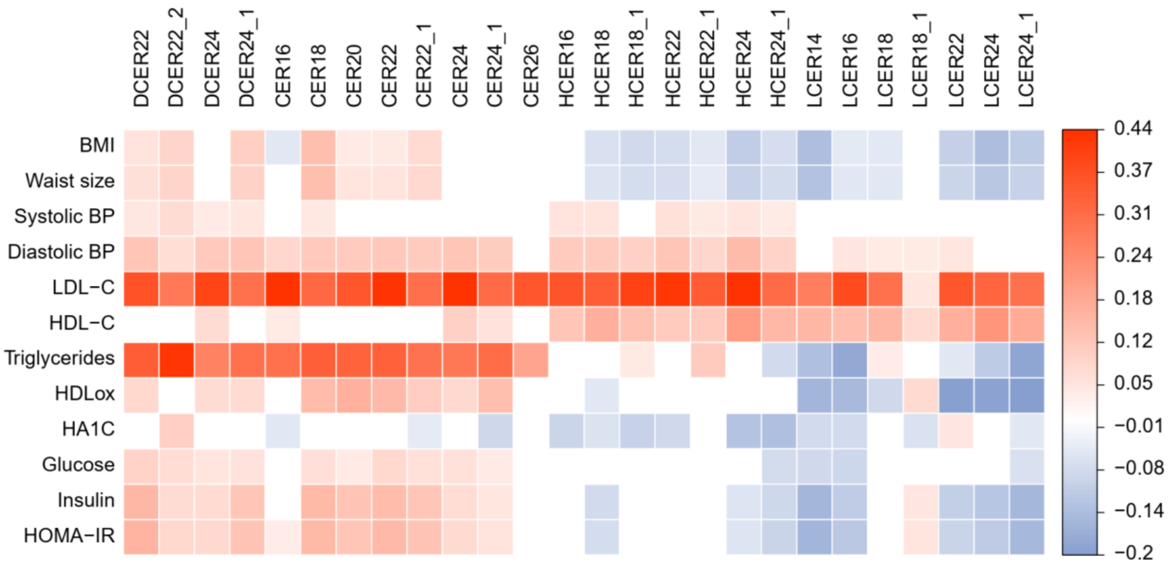

Supplementary Figure 1. Metabolic syndrome-adjusted linear regression of blood sphingolipid species and health biomarkers. Correlation matrix between sphingolipid species and health biomarkers adjusted for sex, age, race, education, and MetS prevalence. Color label represents the regression coefficient of those associations with $p<0.05$ (positive in red, and negative in blue).

Supplementary Table 1. Sociodemographic and health characteristics of the total study sample (n=2,063).

Characteristic	Frequency
Sex	
Men	931 (45.1%)
Women	1,132 (54.9%)
Age (55.7±12.5 years)	
< 50 years	693 (33.6%)
50 - 65 years	894 (43.3%)
>65 years	476 (23.1%)
Race	
Whites	1,543 (74.8%)
African Americans / Blacks	365 (17.7%)
Native Americans	136 (6.6%)
Asian	14 (0.7%)
Others	5 (0.2%)
Educational level	
High school or less	487 (23.6%)
Some college education	1,077 (52.2%)
Postgraduate studies	499 (24.2%)
Nutritional status	
Under weight	11 (0.5%)
Normal weight	494 (23.9%)
Overweight	682 (33.1%)
Obese	876 (42.5%)
Metabolic syndrome	
No	1,291 (62.6%)
Yes	772 (37.4%)
Regular exercise	
No	507 (24.5%)
Yes	1,556 (75,4%)
Smoking status (n=2,060)	
Never	1,164 (56.5%)
Former	627 (30.4%)
Current	269 (13.1%)
Healthy diet index (n=2,051)	
>6.5 (Healthy)	610 (29.7%)
≤6.5 (Unhealthy)	1,441 (70.3%)

Supplementary Table 2. Statistical comparisons of sphingolipid networks and interclasses distances based on the presence of metabolic syndrome and impaired glucoregulation.

		**Interclass distance**		
**Groups**	**Whole net** *p-value*	**DCER-CER** *p-value*	**CER-HCER** *p-value*	**HCER-LCER** *p-value*
$MetS^{-} Gluc^{-}$ vs $MetS^{-} Gluc^{+}$	$1.9x10^{-6}$	ns	ns	ns
$MetS^{+} Gluc^{-}$ vs $MetS^{+} Gluc^{+}$	$<1x10^{-16}$	ns	ns	ns
$MetS^{-} Gluc^{-}$ vs $MetS^{+} Gluc^{-}$	$<1x10^{-16}$	ns	ns	ns
$MetS^{-} Gluc^{-}$ vs $MetS^{+} Gluc^{+}$	$<1x10^{-16}$	ns	ns	0.02
$MetS^{-} Gluc^{+}$ vs $MetS^{+} Gluc^{-}$	$3.1x10^{-11}$	ns	ns	ns
$MetS^{-} Gluc^{+}$ vs $MetS^{+} Gluc^{+}$	$1.2x10^{-12}$	ns	ns	ns

Supplementary Information

Lipid extraction and quantification

Lipid profiling was performed as part of an untargeted-lipidomic approach by Metabolon, Inc. (Durham, NC), following the protocol described below.

- Extraction: Lipids were extracted from the bio-fluid in the presence of deuterated internal standards using an automated BUME extraction according to the method of Lofgren et al[40].

- Data Acquisition: The extracts were dried under nitrogen and reconstituted in ammonium acetate dichloromethane:methanol. The extracts were transferred to vials for infusion-MS analysis, performed on a Shimadzu LC with nano PEEK tubing and the Sciex SelexIon-5500 QTRAP. The samples were analyzed via both positive and negative mode electrospray. The 5500 QTRAP was operated in multiple reaction monitoring (MRM) mode with a total of more than 1,100 MRMs.

- Quantification: Quantitation was built using class-specific internal standards with each class containing at least one, with most containing multiple labeled internal standards. Individual lipid species were quantified by taking the ratio of the signal intensity of each target compound to that of its assigned internal standard, then multiplying by the concentration of internal standard added to the sample (e.g. dCER (d16:0) and dDCER(16:0) for ceramides and dihydroceramides, respectively). Lipid class concentrations were calculated from the sum of all molecular species within a class, and fatty acid compositions were determined by calculating the proportion of each class comprised by individual fatty acids. The quantification of the different species and classes of lipids is reported as micromolar concentration. For our study, we only used sphingolipid values (dihydroceramides, ceramides, sphingomyelins, hexosylceramides -encompassing glucosylceramides and galactosylceramides, and lactosylceramides).

- Data processing: Before statistical analyses, sphingolipid levels were $\log_e$-transformed to achieve normal distributions and normalized using z-score. Only those species with <20% of missing values (due to levels below the lower limit of detection) were included in the statistical modeling.

- Lipidomic data was deposited to OSF database under Accession Name SPLMIDUS_3210391.
